# Psychological rehabilitation of women with breast diseases: dynamics of quality of life before and after a psychotherapeutic program

**DOI:** 10.3389/fpsyg.2026.1795902

**Published:** 2026-04-29

**Authors:** Aliya Uteulova, Ayazhan Janbayeva, Marat Assimov, Tatyana Vasko, Sanzhar Ismailov

**Affiliations:** 1 Kazakh Institute of Oncology and Radiology, Almaty, Kazakhstan; 2 Department of Psychosocial Support, Turan University, Almaty, Kazakhstan; 3 Department of Psychological and Social Support, Kazakh Research Institute of Oncology and Radiology, Almaty, Kazakhstan

**Keywords:** art therapy, body-oriented psychotherapy, breast diseases, oncology, psychology, quality of life, rehabilitation, social adaptation

## Abstract

Breast cancer remains the most prevalent malignant neoplasm among women and is one of the leading causes of cancer-related mortality. Surgical interventions (particularly mastectomy), chemotherapy, and radiotherapy are frequently associated with substantial psychological distress and deterioration in social functioning and overall quality of life. The present study aimed to examine changes in quality of life among women undergoing treatment for breast diseases across different stages of treatment and rehabilitation, as well as to assess changes associated with psychotherapeutic interventions combining individual and group psychological counseling with art therapy and body-oriented psychotherapy techniques. The study included 60 female patients who completed the WHOQOL-BREF questionnaire at three time points: prior to treatment, after treatment, and during the rehabilitation phase. Statistical analysis revealed significant improvements in microsocial support (*p* = 0.010) and social well-being (*p* = 0.010) during the rehabilitation period, while physical and psychological wellbeing remained stable. The impact of disease stage on quality-of-life outcomes was also investigated. The results indicated that disease stage was negatively associated with self-perception (*ρ* = −0.300, *p* = 0.024), microsocial support (*ρ* = −0.372, *p* = 0.004), and social wellbeing (ρ = −0.268, *p* = 0.044). These findings suggest that psychological therapy, delivered through individual and group formats and incorporating art therapy and body-oriented psychotherapy, may be associated with improvements in psychological resilience and social adaptation, particularly in women undergoing treatment for breast diseases.

## Introduction

Breast cancer remains the most prevalent malignant disease among women worldwide and is one of the leading causes of cancer-related mortality. According to the World Health Organization, approximately one million new cases of breast cancer are diagnosed globally each year, with more than 50,000 cases reported annually (Mohammadzadeh et al., 2022). The diagnosis and subsequent treatment of breast cancer, particularly surgical procedures such as mastectomy, represent not only a substantial physical burden but also a profound psychological trauma. The loss of the breast, a powerful symbol of femininity, may significantly affect women’s self-perception and emotional wellbeing. Previous studies indicate that up to 25% of women develop depressive symptoms following mastectomy, while various forms of autonomic dysfunction (ICD-10 F45) are observed in 85–92% of patients (Álvarez-Pardo et al., 2018).

The psychological burden associated with breast cancer extends far beyond the immediate postoperative period. For many patients, the term “tumor” is perceived as synonymous with fatality, evoking feelings of helplessness and existential fear (Volodin et al., 2005). Emotional distress commonly manifests as anxiety, depression, irritability, and psychosomatic symptoms, including dyspnea, tachycardia, gastrointestinal disturbances, and diffuse pain syndromes. Emotional lability, characterized by alternating forced cheerfulness and irritability, is a frequent response pattern. Such psychological instability may compromise treatment adherence, impair social functioning, and reduce overall quality of life (Gokce and Gok, 2022).

Psychosocial maladaptation following mastectomy is a complex and multifactorial process that often exceeds the emotional consequences associated with disability caused by other medical conditions. Disturbances in body image and self-concept negatively influence interpersonal relationships, social participation, and confidence in recovery (Ochoa-Arnedo et al., 2022). Consequently, the psychological dimension of rehabilitation becomes a critical determinant of treatment outcomes. Numerous studies emphasize that effective oncological rehabilitation requires a multidisciplinary approach that integrates medical, psychotherapeutic, and psychoeducational interventions to support emotional and social adjustment (von Au et al., 2024).

One of the most promising directions in contemporary psycho-oncological care is the integration of art therapy and body-oriented therapeutic approaches into rehabilitation programs. Art therapy enables patients to externalize and process traumatic experiences through creative expression, facilitating the integration of suppressed emotions and the reconstruction of a positive self-concept (Gigineyshvili et al., 2019). Creative activity engages kinesthetic, perceptual, and cognitive processes, allowing patients to reinterpret past experiences, construct new meanings, and develop adaptive coping strategies (Czamanski-Cohen et al., 2020). Complementarily, body-oriented psychotherapy techniques—such as breathing exercises, work with muscular tension, and somatic awareness practices—serve as effective tools for psychophysiological stabilization (Guo et al., 2025; Grossert et al., 2017). These approaches reduce chronic tension, regulate anxiety and stress, restore bodily self-awareness, and provide a safe framework for processing trauma related to the cancer diagnosis and surgical intervention.

Given the profound influence of psychological factors on recovery, assessing quality-of-life dynamics and implementing psychotherapeutic interventions are essential components of comprehensive rehabilitation following breast cancer surgery. Therefore, the present study aimed to investigate changes in quality of life among women with breast diseases across different stages of treatment and rehabilitation, as well as to evaluate the effectiveness of an integrative psychotherapeutic program combining art therapy and body-oriented psychotherapy in enhancing psychological resilience, self-perception, and social adaptation.

## Methods

As part of the psychotherapeutic component of the study, a structured psychological intervention program was implemented before treatment, after treatment, and during the rehabilitation period. The program aimed to reduce emotional distress, enhance self-perception, and improve psychosocial adaptation among women diagnosed with breast diseases. Body-oriented psychotherapy techniques were applied in combination with art therapy methods.

The study was designed as a prospective longitudinal quasi-experimental study with repeated measures to assess changes in quality-of-life indicators at three time points. The psychological intervention program was administered at three stages:

1. prior to the initiation of medical treatment (baseline assessment),
2. immediately after completion of treatment (post-treatment assessment),
3. during the rehabilitation phase, 21 days after treatment completion (follow-up assessment).

A total of 60 women with breast diseases participated in the study. The study included women treated for breast pathology at an oncological medical center. The diagnostic spectrum included both malignant (ICD-10 C50) and benign breast conditions (e.g., N63, D24). Patients underwent clinical examination and treatment within the same medical setting and experienced comparable stages of medical treatment and rehabilitation.

Completion of the treatment phase included standard oncological procedures such as surgical treatment (including mastectomy or breast-conserving surgery), as well as chemotherapy and/or radiotherapy according to clinical indications.

### Inclusion criteria

The inclusion criteria for participation in the study were as follows:

- women aged 30–60 years diagnosed with breast diseases (including malignant and benign breast pathology);
- provision of written informed consent;
- patients undergoing preoperative or postoperative stages of treatment.

### Non-inclusion criteria

- Participants were not included in the study if they met any of the following criteria:
- women younger than 30 years or older than 60 years;
- absence of a confirmed breast pathology diagnosis;
- incomplete case report form (CRF) data;
- lack of informed consent;
- diagnosed psychiatric disorders or severe mental pathology.

### Exclusion criteria

Participants were excluded from the study under the following conditions:

- withdrawal from the study at the participant’s request;
- change of country or region of residence during the study period;
- death of the participant before completion of the study;

Quality of life was assessed using the Russian-language version of the WHOQOL-BREF questionnaire. The instrument consists of 26 items rated on a five-point Likert scale.

In the present study, items were grouped into four domains reflecting key aspects of quality of life:

- Physical and psychological wellbeing (items Q3, Q4, Q10, Q15, Q16, Q17, Q18);
- Self-perception (items Q5, Q6, Q7, Q11, Q19, Q26);
- Microsocial support (items Q20, Q21, Q22);
- Social wellbeing (items Q8, Q9, Q12, Q13, Q14, Q23, Q24, Q25).

For negatively worded items (Q3, Q4, Q26), reverse coding was applied prior to score calculation. Domain scores were calculated as the sum of the corresponding item responses.

Internal consistency reliability of the domains was assessed using Cronbach’s alpha coefficients, which demonstrated acceptable to high reliability for all scales (*α* ranging from 0.747 to 0.847).

The psychotherapeutic intervention consisted of structured sessions conducted at three stages of the treatment trajectory: prior to the initiation of medical treatment, immediately after completion of treatment, and during the rehabilitation phase approximately 21 days after treatment completion. The program included three sessions in total, one session at each stage. Each session lasted approximately 60–90 min.

Sessions were delivered either individually or in small groups consisting of 4–6 participants and were facilitated by one clinical psychologist.

The intervention was delivered by qualified clinical psychologists with experience in psycho-oncological support. The art therapy component of the program was conducted by a clinical psychologist certified in art therapy. Body-oriented psychotherapy techniques were delivered by a clinical psychologist with professional experience in body-oriented therapeutic approaches and a Master’s degree in psychological sciences.

Each session followed a structured format consisting of three main components. The first phase included somatic regulation techniques such as diaphragmatic breathing, grounding exercises, guided body scanning, and practices aimed at reducing muscular tension based on Reichian and Lowenian body-oriented psychotherapy approaches.

The second phase consisted of expressive art therapy activities including drawing, collage creation, and body-mapping tasks that enabled participants to symbolically express emotional experiences related to illness and recovery.

The final phase involved verbal reflection and group discussion aimed at integrating bodily sensations with symbolic representations, facilitating emotional processing and cognitive meaning-making.

To clarify the temporal sequence of the study procedures, at each stage participants first completed the WHOQOL-BREF questionnaire, after which the psychological session was conducted. Therefore, the quality-of-life assessments reflected the participants’ condition prior to the intervention at each stage rather than acute post-session effects.

## Results

### Sociodemographic and clinical characteristics

The study included 60 female patients diagnosed with breast diseases. The majority of participants were aged 41–50 years (35.7%), followed by 31–40 years (21.4%) and 51–60 years (17.9%).

Regarding clinical diagnosis, the predominant form was C50.4 (78.3%), while other subtypes (C50.1, C50.2, C50.5, N63, D24) occurred less frequently (each <7%).

- C50.1—Malignant neoplasm of the central portion of the breast (3.3%).
- C50.2—Malignant neoplasm of the upper inner quadrant of the breast (3.3%).
- C50.5—Malignant neoplasm of the lower outer quadrant of the breast (6.7%).
- N63—Unspecified lump in the breast (5.0%).
- D24—Benign neoplasm of the breast (1.7%).

The statistical analysis of the dynamics of psychological indicators across the three stages of the study revealed heterogeneous changes (Table 1). To improve the accuracy of significance estimates, the Monte Carlo method with 95% confidence intervals was applied.

**Table 1:** Dynamics of quality of life domains at different stages of treatment and rehabilitation.

Indicator	Stage 1 median (Q1–Q3)	Stage 2 median (Q1–Q3)	Stage 3 median (Q1–Q3)	Friedman χ^2^ (*p*)	Effect size (*r*)
Physical and psychological wellbeing	24.00 (21.00–26.00)	24.50 (22.00–26.00)	24.00 (22.25–25.00)	1.418 (*p* = 0.502)	–
Self-perception	22.00 (19.00–23.00)	22.00 (20.00–23.00)	22.50 (21.25–25.00)	5.645 (*p* = 0.059)	0.24; 0.22
Microsocial support	11.00 (9.00–12.00)	11.00 (9.00–12.75)	12.00 (10.00–13.00)	9.301 (*p* = 0.010)	0.18
Social wellbeing	30.00 (28.00–32.00)	29.00 (27.00–32.00)	32.00 (28.25–34.00)	9.313 (*p* = 0.009)	0.25

For the physical and psychological wellbeing indicator, no statistically significant changes were observed throughout the study period (Friedman test: χ^2^ = 1.418, *p* = 0.502). Median values remained stable across all three stages of observation (Table 1).

For self-perception, a trend toward statistical significance was identified (χ^2^ = 5.645, *p* = 0.059). Pairwise comparisons demonstrated a significant increase in scores at Stage 3 compared to both Stage 1 (*Z* = −2.633, *p* = 0.009) and Stage 2 (*Z* = −2.424, *p* = 0.015). The effect sizes for these changes were *r* = 0.24 and *r* = 0.22, respectively, indicating small effects.

Analysis of microsocial support revealed statistically significant differences according to the Friedman test (χ^2^ = 9.301, *p* = 0.010). Median values increased from 11.00 at the first two stages to 12.00 at Stage 3. However, pairwise comparisons with Bonferroni correction did not reach statistical significance. The comparison closest to significance was observed between Stage 3 and Stage 2 (*p* = 0.051), which exceeded the adjusted threshold (*α* = 0.017). The comparison between Stage 3 and Stage 1 also did not reveal significant differences (*p* = 0.171). These findings may indicate that the overall trend is significant, while differences between specific stages remain modest and are not detected under the conservative Bonferroni correction. The effect size for the comparison between Stage 3 and Stage 2 was *r* = 0.18, indicating a small effect.

The most pronounced dynamics were observed for social wellbeing. The Friedman test confirmed statistically significant differences between the three measurements (χ^2^ = 9.313, *p* = 0.009). Pairwise comparisons showed that the significant increase occurred between Stage 2 and Stage 3 (*Z* = −2.707, *p* = 0.007). The median increased from 29.00 at Stage 2 to 32.00 at Stage 3. The effect size for this change was *r* = 0.25, corresponding to a small effect approaching a medium magnitude. Differences between Stage 1 and Stage 3 did not reach the corrected level of statistical significance (*p* = 0.077), indicating only a trend toward improvement.

Thus, the study revealed statistically significant positive changes in self-perception and social wellbeing, primarily occurring between the second and third stages of the study. The narrow confidence intervals for significant and near-significant effects (for example, 0.005–0.011 for social wellbeing) indicate a high level of precision and reliability of the *p*-value estimates obtained using the Monte Carlo method.

For microsocial support, although the overall Friedman test was statistically significant, pairwise comparisons with Bonferroni correction did not reach statistical significance, indicating that the observed changes should be interpreted with caution. Physical and psychological wellbeing remained stable throughout the entire study period.

The observed effect sizes (*r* = 0.22–0.25) were interpreted as small, suggesting the presence of positive trends that may require a longer observation period or a larger sample size for more robust confirmation.

The correlation analysis conducted using Spearman’s rank coefficient did not reveal any statistically significant associations between the level of education and the four quality-of-life domains (physical and psychological wellbeing, self-perception, microsocial support, and social wellbeing) across all three stages of the study (*p* > 0.05) (Table 2).

**Table 2:** Correlations between education level and quality of life domains at different stages of treatment.

Quality of life domain	Stage 1	*p*-value	Stage 2	*p*-value	Stage 3	*p*-value
Physical and psychological wellbeing	0.042	0.748	0.186	0.154	−0.117	0.372
Self-perception	0.150	0.253	−0.004	0.973	−0.012	0.926
Microsocial support	0.164	0.210	0.171	0.192	0.120	0.360
Social wellbeing	0.238	0.067	0.062	0.635	0.100	0.448

The majority of correlation coefficients were weak and positive, particularly at Stage 1, and gradually approached zero across subsequent stages. The only trend approaching statistical significance was observed for social wellbeing at Stage 1 (*ρ* = 0.238, *p* = 0.067), indicating a potential initial advantage in social adaptation among patients with higher educational attainment, although this effect did not reach statistical significance. Overall, the findings suggest that educational level does not exert a significant influence on quality-of-life outcomes in women with breast disease at any stage of treatment or rehabilitation. Accordingly, psychoeducational and psychotherapeutic interventions may be applied effectively regardless of patients’ educational background, without the need for differentiated intervention strategies.

Correlation analysis identified several statistically significant negative associations between disease stage and specific quality-of-life domains (Table 3).

**Table 3:** Сorrelations between disease stage and quality of life domains at different stages of treatment.

Quality of life domain	Stage 1	*p*-value	Stage 2	*p*-value	Stage 3	*p*-value
Physical and psychological wellbeing	−0.177	0.187	−0.151	0.264	−0.019	0.889
Self-perception	−0.168	0.213	−0.300*	0.024	−0.229	0.087
Microsocial support	0.120	0.372	−0.157	0.244	−0.372	0.004
Social wellbeing	−0.045	0.741	−0.051	0.704	−0.268	0.044

All coefficients were calculated using Spearman’s rank correlation. Significance levels: *p* < 0.05 (*), *p* < 0.01 (**). Negative values indicate an inverse relationship between disease stage and quality-of-life indicators.

At the post-treatment stage (Stage 2), disease stage was moderately negatively correlated with self-perception (*ρ* = −0.300, *p* = 0.024), indicating lower levels of self-acceptance and psychological adjustment among patients with more advanced disease immediately after treatment. During the rehabilitation phase (Stage 3), significant negative correlations were found for microsocial support (*ρ* = −0.372, *p* = 0.004) and social wellbeing (ρ = − 0.268, *p* = 0.044), suggesting that patients with higher disease stages experience greater difficulties in social adaptation and perceive reduced support from their immediate social environment. No significant correlations were observed at the pre-treatment baseline. This pattern indicates that the impact of disease stage intensifies over time and becomes most pronounced during rehabilitation. These findings highlight the need for targeted psychotherapeutic interventions aimed at strengthening microsocial and social functioning in patients with advanced disease, as well as timely psychological support to enhance self-perception following medical treatment.

## Discussion

The present study investigated changes in quality-of-life indicators among women with breast disease across different stages of treatment and rehabilitation and examined the influence of educational level and disease stage on psychosocial adaptation. The results contribute to the growing body of evidence emphasizing the importance of psychological rehabilitation and integrative therapeutic approaches in oncology.

WHOQOL-BREF assessment revealed domain-specific changes in quality of life. Physical and psychological wellbeing remained stable throughout the study period, suggesting that medical treatment and subsequent rehabilitation did not lead to substantial deterioration or improvement in physical functioning. This stability may reflect effective medical management and patients’ resilience in maintaining functional equilibrium during treatment.

In contrast, self-perception, microsocial support, and social wellbeing demonstrated positive trends during the rehabilitation phase. The most pronounced improvements were observed in microsocial support and social wellbeing, where statistically significant increases were recorded during the rehabilitation period. These findings are consistent with previous studies indicating that psychosocial and group-based interventions enhance perceived social support, self-efficacy, and social integration. The observed improvements may be associated with the psychotherapeutic program implemented in this study, although they may also reflect natural psychological adaptation processes during recovery. Such integrative approaches have been shown to activate adaptive mechanisms and foster psychological resilience.

No statistically significant correlations were found between educational level and any quality-of-life domains (*p* > 0.05) across all stages of the study. Although a weak positive trend was observed between education and social wellbeing at baseline (*ρ* = 0.238, *p* = 0.067), this association was not statistically confirmed. These findings indicate that educational level is not a predictive factor for quality-of-life outcomes in women with breast disease, aligning with previous research suggesting that psychosocial responses to illness are more strongly influenced by coping strategies, emotional regulation, and social support than by educational attainment. Therefore, psychoeducational and psychotherapeutic interventions can be equally effective across different educational groups without the need for academic-level stratification.

In contrast, disease stage showed significant associations with several psychosocial dimensions. At the post-treatment stage, more advanced disease was associated with lower self-perception (*ρ* = −0.300, *p* = 0.024), indicating reduced self-acceptance and emotional adjustment immediately after surgery. During rehabilitation, significant negative correlations were observed between disease stage and microsocial support (ρ = −0.372, *p* = 0.004) as well as social wellbeing (ρ = −0.268, *p* = 0.044). These findings suggest that patients with more advanced disease experience greater difficulties in social adaptation and perceive less interpersonal support, potentially due to prolonged physical limitations, fear of recurrence, and social withdrawal. The increasing strength of these associations at later stages indicates that the impact of disease severity becomes more pronounced during long-term recovery, underscoring the necessity of targeted interventions focused on restoring social functioning and strengthening supportive environments for patients with advanced disease.

The integration of a psychological program combining body-oriented psychotherapy and art therapy constituted a valuable component of the rehabilitation process. Body-oriented therapy, through the integration of bodily sensations and symbolic representations, facilitated emotional processing, cognitive meaning-making, and stabilization of physiological and emotional states, while art therapy enabled participants to express illness-related emotions and reconstruct a positive self-image. Together, these methods supported a more holistic recovery process, enhancing both emotional regulation and social adaptation. The observed positive trends in microsocial and social wellbeing provide preliminary support for incorporating creative and body-oriented techniques into psycho-oncological rehabilitation programs.

Previous studies have demonstrated that art therapy interventions can reduce emotional distress and improve psychological wellbeing in oncology patients (Monti et al., 2006; Thyme et al., 2009). Similarly, body-oriented psychotherapy approaches have been associated with improved emotional regulation and reduced somatic tension (Röhricht, 2009).

Despite the significance of the findings, several limitations should be acknowledged. The study involved a relatively small sample size (*N* = 60) and was restricted to a specific clinical population within a single medical institution. Future research should expand the sample and include longitudinal follow-up assessments to evaluate the long-term sustainability of the observed effects. Additionally, incorporating qualitative interviews could provide deeper insights into subjective experiences and individual differences in coping mechanisms.

One important limitation of the present study is the absence of a control group, which restricts the ability to attribute the observed improvements in microsocial support and social wellbeing exclusively to the psychotherapeutic intervention. These changes may partly reflect natural psychological adaptation processes occurring during recovery from cancer treatment. Future studies should incorporate control groups and larger samples to provide a more rigorous evaluation of psychotherapeutic rehabilitation programs.

In addition, socio-demographic characteristics such as marital status, income level, and broader socioeconomic conditions may influence perceived microsocial support and social wellbeing. Future research should consider these variables to better understand their potential role in psychosocial adaptation among women with breast disease.

## Conclusion

The present study indicates that an integrative psychotherapeutic intervention combining body-oriented techniques and art therapy may be associated with improvements in self-perception and social wellbeing among women with breast diseases during the rehabilitation process.

Although the observed effect sizes were small, the findings suggest that psychosocial interventions may support emotional adaptation during cancer recovery. However, the absence of a control group and the relatively small sample size limit causal interpretation. Future controlled studies are required to more rigorously evaluate the effectiveness of psychotherapeutic rehabilitation programs in oncological populations.

## Data Availability

The original contributions presented in the study are included in the article/supplementary material, further inquiries can be directed to the corresponding author.
